# Maternal Stressors and Coping Strategies During the Extended Postpartum Period: A Retrospective Analysis with Contemporary Implications

**DOI:** 10.1089/whr.2021.0134

**Published:** 2022-01-31

**Authors:** Lorraine O. Walker, Nicole Murry

**Affiliations:** The University of Texas at Austin, School of Nursing, Austin, Texas, USA.

**Keywords:** coping, employment, postpartum, stress

## Abstract

***Background:*** Despite recent emphasis on the “fourth trimester” and beyond, most knowledge of stressors affecting women is focused on the first 6 postpartum weeks. Our aim was to identify postpartum-specific stressors and coping over the extended postpartum period.

***Methods:*** We analyzed data from two surveys for a combined sample of 346 postpartum women. Principal components analysis of survey items on sources of stress was used to identify categories of postpartum-specific stressors. Content analysis was used to categorize text data on coping strategies.

***Results:*** Seven stressors were identified: Overload, Working mother concerns, Isolated motherhood, Limited supportive resources, Exhaustion, Parenting demands, and Changes in body and sexuality. Overload was the most frequent stressor (*F* = 49.32, *p* < 0.001) and was significantly higher at 9–12 months than at 5–8 months or at 13 months or more (*F* = 6.42, *p* = 0.002). Fulltime employment and having more than one child were associated with elevated scores on several stressors. Content analysis yielded seven coping strategies, such as Take time alone or with others, Manage emotions and thoughts, and Maintain a manageable workload. Five of the seven stressors were associated with at least one of the top five coping strategies; none was associated with Overload or Limited supportive resources.

***Conclusions:*** Women's predominant source of stress was from overload and was highest at 9 to 12 months postpartum. Community resources and public health policy and programming are needed to prepare and support women during the challenging first postpartum year.

## Introduction

Motherhood is not necessarily an easy transition and brings stress, whether having a first or a subsequent child. Underlying structural factors, such as lack of national paid maternity leave policy in the United States, may contribute to women's stress.^1,2^

Although studies have documented postpartum stressors, most have focused on the first 6 postpartum weeks. That period has been superseded by the “fourth trimester.”^3,4^ More recently, key sources^5–7^ called for further extending coverage for the first postpartum year to reduce maternal morbidities and mortality. This extended view of the postpartum period is supported by evidence that postpartum stress persists during and beyond the first postpartum year.^8,9^ Thus, a probe into women's specific stressors and coping strategies during the extended postpartum period is warranted for more informed health care and support of women.

Stress is classically defined as “situations in which the demands on individuals tax or exceed their adaptive capabilities.”^10^^(p.3)^ Postpartum stress is particularly important because it is one of the key factors associated with postpartum depression.^11,12^ If untreated, depression has multiple negative effects on women, such as functional and relationship difficulties and reduced quality of life.^13^ Postpartum stress is also linked to women's health behaviors, including less healthy diet, smoking relapse, and postpartum weight retention.^14–16^

Early studies of postpartum stress identified women's top stressors during the early postpartum weeks, such as sleep disturbances and fatigue.^17–19^ Horowitz and Damato showed that stressors at 6-week postpartum fell into four broad categories: roles, tasks, resources, and relationships.^18^ A recent study found similar findings with the addition of child health concerns.^20^ By delving into postpartum-specific stressors, the preceding studies give texture to the challenges faced by women compared to studies using stress scales for the general population. A similar point about the need for postpartum-specific measures was made by Razurel et al.^21^ Textured knowledge about postpartum-specific stressors is essential for health care providers (HPCs) to provide meaningful parent counseling.

Although knowledge of stressors extending through the first postpartum year is needed, only a few studies have addressed that period. Two such studies showed that women's stress was higher at 12 months postpartum than earlier,^8,9^ but whether these differences were statistically significant was unclear. Contextual factors, such as employment, education, and number of children, also may affect women's postpartum stress.^2,22,23^ Finally, because an extensive list of stressors is challenging to interpret and apply, categorizing related postpartum-specific stressors provides a means of increasing their usefulness.

Closely related to stressors is how women manage them, which falls under the rubric of coping: “how individual efforts to manage distressing problems and emotions affect the physical and psychological outcomes of stress.”^24^^(p.620)^ Most studies of postpartum coping have focused on predicting women's mental health; most have used scales for the general population without adaptation to the postpartum context.^25–27^ Thus, broad coping categories were typical foci of such studies.

Only a few studies have covered women's postpartum-specific coping strategies. One study found women's top coping strategy after hospital discharge was mobilizing internal resources.^28^ In another, women used being planful, getting help, and taking breaks to maintain wellness during the first postpartum year.^29^ Much less is known about how postpartum-specific stressors are associated with coping strategies during the postpartum period, and whether sources of stressors vary over time.

In this study, we used archival data gathered before 2000, which focused on postpartum-specific stressors and coping strategies during the extended postpartum period. Our overall goal was to delineate such stressors and coping and consider if there is evidence in the literature that such stressors are continuing to affect women's lives.

To gain insights into postpartum-specific stressors and coping during this understudied period, our aims were (1) to examine categories of women's postpartum-specific stressors during the extended postpartum period and associations with contextual factors (postpartum time points, employment status, number of children, and maternal education); (2) to identify categories of postpartum-specific coping, and (3) to explore the association of stressors and coping strategies during the extended first postpartum year.

## Methods

### Design and data sources

This study is based on archival de-identified data from two longitudinal surveys of women's health, behaviors, and stress during the extended postpartum period: a Central Texas stratified random sample^30^ and a Midwestern systematically sampled cohort.^31^ We used Central Texas data from women at 8 to 18 months postpartum and Midwestern data from two cohorts at 6 and 12 months postpartum, respectively. Names and addresses of women were drawn from newspaper birth announcements.

Where both samples shared the same variables, data were merged to enhance sample size for certain statistical analysis. Other analyses drew on data from the Central Texas sample only, as described further in the Data Analysis section. The final analytic sample was 346 women after merging samples and deleting 10 cases with no stress data and one case younger than 18 years. Institutional Research Board approvals were obtained in accord with existing university procedures.

### Participants

Characteristics of the study samples are provided in Table 1. Women in the samples were predominantly White and married with some college education. Mean age across the samples was in the late 20s. Women were between 5 and 20 months postpartum, with 123 women between 5 and 8 months postpartum, 154 between 9 and 12 months, and 69 at 13 months or more.

**Table 1.** Characteristics of Central Texas and Midwestern Samples

	Central Texas, ***n*** = 119, ***n (%)***	Midwestern: 12 months cohort ***n*** = 105, ***n (%)***	Midwestern: 6 months cohort ***n*** = 122, ***n (%)***
Months postpartum when stress measured, *mean (SD, range)*	13.3 (3.3, 8–20)	11.8 (0.6, 11–14)	5.9 (0.5, 5–8)
Maternal age at first panel in years, *mean (SD, range)*	29.0 (4.1, 18–40)	28.5 (5.1, 18–40)^a^	27.3 (4.5, 18–38)^a^
Race/ethnicity			
White (non-Hispanic)	107 (89.9)	105 (100)	116 (95.1)
Black/African American	0 (0)	0 (0)	2 (1.6)
Hispanic or Mexican	12 (10.1)	0 (0)	2 (1.6)
Asian	0 (0)	0 (0)	2 (1.6)
Employment status			
Not employed	50 (42.0)	48 (45.7)	63 (51.6)
Part-time employed	17 (14.3)	27 (25.7)	29 (23.8)
Full-time employed	45 (37.8)	30 (28.6)	30 (24.6)
Other, worked from home	7 (5.9)	0 (0)	0 (0)
Number children			
1	66 (55.5)	39 (37.5)^a^	41 (33.9)^a^
2	33 (27.7)	36 (34.6)	51 (42.1)
3 or more	20 (16.8)	29 (27.9)	29 (24.0)
Married	115 (96.6)	102 (98.1)^a^	121 (99.2)
Ever attended college or university	96 (82.1)^a^	62 (59.0)	74 (60.7)

^a^ Missing data on 1 or more cases.

SD, standard deviation.

### Measures and procedures

The Sources of Stress Scale (SoS), a 27-item scale, was used to measure postpartum stressors of women over the last month.^32^ SoS items drew on earlier responses of the Central Texas sample to a question at 2 to 12 months postpartum: “Based on your experience, what do you feel are the two main causes of stress for mothers with babies?” Those responses formed the basis for developing SoS items. SoS response options are on a 5-point scale: 0 = Never, 1 = Almost Never, 2 = Sometimes, 3 = Fairly Often, and 4 = Very Often. The SoS has a Cronbach α of 0.87, and validity is supported by a correlation of 0.63^30^ with the well-established Perceived Stress Scale.^33,34^ The SoS was available from both the Central Texas and Midwestern samples.

Coping strategies—gathered in the Central Texas sample at the same time as the SoS responses—were derived from responses to the following question: “Based on your experiences as a mother, what have you found to be helpful ways to manage the stresses of parenting?” This question was followed by space for two written answers. Responses were categorized by content analysis (see Data Analysis section).

Demographic information in survey questionnaires included the following: infant age at the time of completing the survey forms (postpartum time point), employment status, race/ethnicity, and maternal education (whether ever attended a college or university). Because of ambiguous wording of a breastfeeding item, data on that variable were excluded.

### Data analysis

Descriptive analysis included examination of frequencies and percentages for nominal data, and means, standard deviations (*SD*s), ranges, and normality of continuous data.

Principal components analysis (PCA) was used to group SoS stressors into categories. SoS data from the Central Texas and Midwestern samples were merged to provide a robust sample size for the PCA. Merging resulted in a combined sample size that met the criteria of having at least a 1:10 ratio of variables to cases and having at least 300 cases.^35,36^ The choice of PCA was made because items about stressors were not derived from a predefined theoretical concept,^35^ but rather from stressors identified by women. Applicability of data for PCA was supported by the Kaiser–Meyer–Olkin (KMO) measure of sample adequacy (KMO = 0.843) and Bartlett's test of sphericity (χ^2^ = 3032.4, *df* = 325, *p* = 0.001).

Eigenvalues of 1.0 or higher and interpretability were used to determine the number of components extracted. Rotated component loadings were based on varimax rotation. One item on breastfeeding problems was positively skewed and interfered with reaching convergence on rotated components, so it was omitted from the PCA analysis, and treated as a separate stressor because of the importance of breastfeeding.

Since scores based on PCA loadings on components are sample specific, we instead used summated means for the stressor categories to increase comparability with future studies. Furthermore, to enable comparisons of means across stressor categories, we adjusted for the number of items in a category. For example, a category of 5 items with a total mean of 15.5 would be divided by 5 to yield an adjusted category mean of 3.1. Cronbach α values were computed for PCA categories.

Differences among adjusted means of stressor categories were examined by repeated measures analysis of variance (ANOVA) with the *F* tests based on the Greenhouse–Geisser correction.^37^ Differences in adjusted means by contextual factors were tested using one-way ANOVA with the robust Brown–Forsythe *F* test, because some comparisons revealed significant differences in homogeneity of variances among groups.^37^ For the same reason, the Games–Howell procedure was used in *post hoc* pairwise comparisons.

Content analysis^38,39^ was used to develop categories of coping strategies in the Central Texas sample. The coding guide was developed by both authors using an inductive process. First, after reading women's responses, each author independently developed preliminary codes. These were then compared, and adjustments were made to develop a final coding scheme. Each author then independently coded the coping responses provided by women.

The Kappa statistic, a measure of inter-rater reliability, was 0.81 (*p* < 0.001) and 0.71 (*p* < 0.001), for the first and second coping strategies that mothers reported, respectively. To explore associations between stressor categories and coping strategies, which were dichotomous, we computed point-biserial correlations between the stressor categories and the first and then second coping strategies. Statistical significance was set at 0.05.

## Results

### Stressor categories

Table 2 presents rotated component loadings from the PCA analysis and Cronbach α values for categories. Seven components were extracted and labeled: (1) Overload, (2) Working mother concerns, (3) Isolated motherhood, (4) Limited supportive resources, (5) Exhaustion, (6) Parenting demands, and (7) Changes in body and sexuality. Adjusted means ranged from 2.3 to 1.39 (Table 3). However, for each component, a minority of women had adjusted scores of 3 or higher, corresponding to “Fairly often” to “Very often.”

**Table 2.** Principal Component Analysis: Stressors by the Component on Which Each Loaded the Highest

Principal components	Loadings of items on components							α^a^
1: Overload								0.82
9. Lack of time to do things that are personally enjoyable to you?	0.701							
3. Too many household responsibilities?	0.657							
6. Not enough time for husband/partner?	0.626							
8. Too many family responsibilities?	0.619							
10. Lack of time alone?	0.599							
2: Working mother concerns								0.74
2. Unhappiness/worry about working outside the home, while your child is an infant?		0.808						
23. Feeling bad when you have to leave the baby?		0.716						
5. Not enough time for other responsibilities with working outside of your home?		0.644						
12. Concern about the quality of the babysitter or childcare arrangements for your baby?		0.629						
1. Baby's illness or concern about your baby's health?		0.426						
3: Isolated motherhood								0.73
11. Too much time alone with your baby?			0.717					
21. Changes in your lifestyle since the baby came?			0.664					
19. Feeling that you do not know what your baby wants or needs?			0.629					
4. Having round the clock responsibility for your baby?			0.542					
20. Feeling depressed or useless?		.	0.507					
4: Limited supportive resources								0.59
17. Not enough help from husband with baby and home responsibilities?				0.752				
24. Not enough understanding from your husband/partner about your pressures?				0.691				
16. Financial problems?				0.459				
22. Interference from in-laws?				0.408				
5: Exhaustion								0.69
13. Not enough sleep?					0.810			
14. Not enough rest?					0.760			
15. Baby's crying or colic?					0.443			
6: Parenting demands								0.53
7. Not enough time for other children since baby came?						0.771		
25. Being patient with the baby or your other children when you are tired or upset?						0.577		
7: Changes in body and sexuality								0.36
26. Being overweight?							0.723	
27. Changes in sexual patterns?							0.532	

^a^ Cronbach α for summed items.

**Table 3.** Descriptive Statistics for Stressor Categories

Stressor categories	Mean^a^	SD	Median^a^	No. of items
1: Overload	2.30	0.87	2.40	5
2: Working mother concerns	1.68	0.93	1.60	5
3: Isolated motherhood	1.39	0.76	1.40	5
4: Limited supportive resources	1.74	0.83	1.75	4
5: Exhaustion	1.76	0.85	1.67	3
6: Parenting demands	1.60	0.97	1.50	2
7: Changes in body and sexuality	1.82	1.04	2.00	2

^a^ Adjusted for the number of items in a category.

Adjusted means differed significantly among the seven categories (*F*[5.3, 1834.5] = 49.32, *p* < 0.001); the predominant finding was that the mean for Overload was significantly higher than the other six category means (all *p'*s < 0.001). For breastfeeding concerns, the distribution for “never/almost never” was 92.7%, “sometimes” 2.3%, fairly/very often 4.0%, and missing 0.9%.

Postpartum time periods were associated with significant differences on Overload (*F*[2, 266] = 6.42, *p* = 0.002) and Exhaustion (*F*[2, 273] = 3.51, *p* = 0.031). The Overload adjusted mean was significantly higher at 9–12 months (*M* = 2.49, *SD* = 0.82) than at 5–8 months (*M* = 2.16, *SD* = 0.89) or 13 months or more (*M* = 2.15, *SD* = 0.86). The Exhaustion adjusted mean was significantly higher at 9–12 months (*M* = 1.83, *SD* = 0.82) than at 13 months or more (*M* = 1.52, *SD* = 0.83).

Maternal employment was associated with significant differences on adjusted category means for Overload (*F*[2, 281] = 11.49, *p* < 0.001) and Working mother concerns (*F*[2, 250] = 60.91, *p* < 0.001). The Overload adjusted mean was significantly higher for women employed full time (*M* = 2.62, *SD* = 0.80) compared to those employed part time (*M* = 2.23, *SD* = 0.82) or not employed (*M* = 2.12, *SD* = 0.89). For Working mother concerns, all levels of employment status differed significantly as follows: not employed (*M* = 1.23, *SD* = 0.79), employed part time (*M* = 1.78, *SD* = 0.86), and employed full time (*M* = 2.34, *SD* = 0.78).

The number of children was associated with significantly higher adjusted means for Overload (*F*[2, 299] = 7.31, *p* = 0.001), Exhaustion (*F*[2, 268] = 3.80, *p* = 0.024), and Parenting demands (*F*[2, 281] = 86.61, *p* < 0.001). Women with either two children (*M* = 2.42, *SD* = 0.86) or three children or more (*M* = 2.50, *SD* = 0.84) had significantly higher Overload adjusted means compared to those with one child (*M* = 2.10, *SD* = 0.86).

For Exhaustion, women with two children (*M* = 1.87, *SD* = 0.79) had significantly higher adjusted means than those with one child (*M* = 1.62, *SD* = 0.84). For Parenting demands, women with two children (*M* = 2.08, *SD* = 0.90) or three children or more (*M* = 2.12, *SD* = 0.79) had significantly higher adjusted means compared to those with one child (*M* = 0.94, *SD* = 0.69).

Maternal education was associated with differences in Limited supportive resources (*t*[342] = −4.01, *p* < 0.001). Women who never attended college/university had higher stress scores for Limited supportive resources (*M* = 1.99, *SD* = 0.83) than women who ever attended (*M* = 1.62, *SD* = 0.81). Finally, breastfeeding problems were not associated with any contextual factor.

### Coping categories and their association with stressors

Table 4 presents the categories of coping strategies. Of 119 women in the Central Texas sample, 111 reported a first coping strategy and 97 reported a second one. The most frequent first and second coping strategies women reported were the same: take time alone or with others for respite or recovery. The frequency and ranking of the other coping strategies are in Table 5.

**Table 4.** Coding Categories

No.	Categories	Definitions	Examples
1	Engage in health promoting activities	Includes exercising; eating a healthy diet; getting rest and sleep:	Exercise Eat well Sleep and/or rest Take naps when baby asleep
2	Take time alone or with others for respite or recovery	Includes taking time for self; doing personal care; going out alone or with others, but does not mention being social or interacting	Time alone Take evening/weekend out Going out with others; Take a bath
3	Engage in spiritual care	Includes prayer and/or meditation	Prayer; Meditating
4	Engage in social interactions and seek social support	Includes talking and socializing with others; maintain or seek support	Attend support or other groups; seek support from spouse, extended family or others, including fellowship; Talking with spouse, extended family, or others
5	Develop or maintain a manageable workload (for Locus/sense of control)	Includes use of planning, scheduling, organizing within the family; sharing or assigning tasks with others; being flexible; and avoiding overcommitting	Organize and/or schedule routines Get household help; Share tasks, including child care, with spouse Keep older children entertained; Balance planning with flexibility
6	Manage emotions and thoughts	Includes use humor or mental strategies to reframe; or use of techniques to manage emotions	Use Humor, deep breathing Read on parenting Reflection, eg., on preciousness of baby Actively suppress feelings of stress or guilt Remain calm when children upset Accept stress as part of life; Remind self this will pass
7	Other	Unclear or rare strategies	Outside interests; Quit job; Clean; Putting child first

**Table 5.** Frequency and Ranking of First and Second Coping Strategies

Strategy in response to stressors	First strategy		Second strategy	
	Reported frequency	Rank	Reported frequency	Rank
2. Take time alone or with others for respite or recovery	34	1	28	1
5. Develop or maintain a manageable workload	24	2	17	2.5
4. Engage in social interactions and seek social support	21	3	14	4
1. Engage in health promoting activities	15	4	13	5
6. Manage emotions and thoughts	12	5	17	2.5
3. Engage in spiritual care	3	6	0	7
7. Other	2	7	8	6
Total	111		97	

Among the five frequently used first coping strategies, two correlated significantly with stressors. Take time for respite or recovery was negatively correlated (*r*_pb_ = −0.22, *p* = 0.020) with Changes in body and sexuality. Managing emotions and thoughts was positively correlated (*r*_pb_ = 0.20, *p* = 0.036) with Parenting demands.

Among the five frequently used second coping strategies, six correlations with stressors were significant. Taking time for respite or recovery was negatively correlated with Working mother concerns (*r*_pb_ = −0.23, *p* = 0.022) and positively correlated with Isolated motherhood (*r*_pb_ = 0.25, *p* = 0.014). Engage in social interactions and seek social support was positively correlated with Exhaustion (*r*_pb_ = 0.22, *p* = 0.030) and with Breastfeeding problems (*r*_pb_ = 0.34, *p* = 0.001). Engage in health promoting activities was negatively correlated with Changes in body and sexuality (*r*_pb_ = −0.25, *p* = 0.014). Manage emotions and thoughts was positively correlated with Working mother concerns (*r*_pb_ = 0.20, *p* = 0.049).

## Discussion

### Stressors during the extended postpartum period

Our aim was to gain a textured understanding of the stressors and coping strategies specific to the extended postpartum period, which in this study covered 5 to 20 months postpartum. In this study, we consider the nature of postpartum-specific stressors and evidence from the literature that these continue to affect the lives of women with infants.

Overload, defined by having too many responsibilities for the time available, was the leading stressor reported by women in this study. It was highest at 9 to 12 months postpartum, and it continues to be a stressor for women.^1,2,40^ Mothers' caregiving may become more intense around 12 months as infants are mobile; attachment is focused on the main caregiver, but language is yet limited. We found overload was highest for two demographic groups: women who were employed full time and those with more than one child, which increases women's spectrum of responsibilities.

Working mother concerns were defined by concerns about working while one's child is an infant, quality of childcare, and infant illness, and are ones that women continue to express.^22,41^ In this study, concerns increased as women's work involvement increased and are consistent with current literature about women's feelings of not being a good enough mother on returning to work.^22^

Isolated motherhood was defined by too much time alone, loss of prior lifestyle, being unclear about the baby's wants, unrelenting caregiving, and depressive feelings. This stressor continues to affect postpartum women, and often is associated with loss of prior life patterns and contact with others, and with postpartum depression.^1,42^ Isolated motherhood did not differ by contextual variables in this study and is concerning because of its association with women's mental health.^42^

Limited supportive resources were primarily defined by frequently experiencing insufficient help (instrumental support) and understanding (emotional support) from women's spouses/partners, and financial problems. While supportive resources may mitigate stress, if support is low, it may itself become a source of stress.^43^ This source of stress continues to be reported by postpartum women.^44–46^ In this study, women without any college education reported limited supportive resources more often compared to those who had ever attended college. Given that education is an indicator of socioeconomic position, our finding may reflect interconnections among factors related to social disadvantage.^47^

Exhaustion, defined by items about not enough sleep and rest and infant crying, was higher at 9–12 months and is a stressor women continue to report.^1,48,49^ It also was significantly higher among women with two children compared to one. Transitioning from caring for one child compared to two brings new sources of tension and demands^50^ and increases the time required to manage children's needs.

Parenting demands were defined by lack of time for other children with a new baby in the family and concern about being patient when faced with children's needs. Parenting demands were increased among women with two or more children. Parenting stress continues to be reported in association with variables such as family structure, race/ethnicity, and child health.^23,51^

Changes in body and sexuality did not differ by contextual factors, but each continues to be reported by postpartum women.^23,52–54^ It is noteworthy that the association of body image and sexuality is discussed in the popular media,^55^ but is examined in only a few studies.^54,56^

Breastfeeding problems, which were reported by only a small percentage of women in this study, are generally concerns during the early postpartum weeks.^45,48^ We found contextual variables were unrelated to breastfeeding problems. These null findings may stem from the time frame of this study, wherein breastfeeding problems were already resolved.

Overall, the postpartum-specific stressors identified in our archival data continue to be reported in the literature. Many of these stressors are inherently tied to the social, economic, and political context of motherhood.^1,2^ While some resources in that context have changed, such as access to mobile communication and information through the Internet,^48,57^ others have not. The United States still lacks a nationally mandated paid maternity leave. Women face limited access to affordable quality childcare, supportive community resources, home-visiting nurses, and pay equity, while confronting an ethos of high maternal caregiving expectations.^1,2,42^

Despite these challenges, it is also important to note benefits that women derive from employment outside the home. Besides economic rewards, generally, women who are employed enjoy better mental health than women who are not employed.^58^ Women also report job-related rewards, such as helping others, and being challenged and recognized.^59^ However, for mothers of young children, mental health benefits may be tempered by job quality and employment preference.^60^

### Coping strategies and their association with stressor categories

Women's coping strategies ranged from more reactive (*e.g.*, Manage emotions and thoughts) to ones that were more proactive (*e.g.*, Develop or maintain a manageable workload). The postpartum-specific coping strategies identified in this study are similar to ones in the literature,^1,23,29^ with the exception of health promotion strategies. However, the use of such strategies as exercise to manage stress is reported in other populations.^61,62^

The associations we found between stressors and coping strategies support the need for more in-depth study of how women manage stressors in the extended postpartum period. Developing more refined postpartum-specific coping scales would support more complex analyses than our dichotomous measures of coping permitted. Equally important is the finding that no coping strategy was associated with Overload, women's most frequent source of stress. This warrants further exploration, given the centrality of overload in the postpartum period.

Finally, because the period when our data were collected preceded widespread availability of social media as sources of support and communication, these were not evident in the coping categories. However, Engage in social interactions and seek social support may have captured some of the support that women would gain from use of media.

### Implications

While it is already known that the extended postpartum period may be stressful,^9^ understanding when certain postpartum-specific stressors may be prominent and how women cope with them adds textured knowledge needed by HPCs for anticipatory guidance and counseling of postpartum women. With so much attention to the stress of motherhood in the first 6 weeks after birth, longer term coping skills are often overlooked. The burden of patient education and support during the fourth trimester and 1st year after childbirth fall primarily on support in the community.

The findings of this study will help to inform the anticipatory guidance and support provided by HPCs who work with pregnant and postpartum women and their children in both clinical settings and in the community. Patient education on leaving the hospital after giving birth is an important opportunity for discharge nurses to share information, but it is insufficient in providing longer term advisement.^63^ Although postpartum clinical visits are often limited to the traditional 6-week checkup, this is an important opportunity for screening of postpartum women for signs of stress by HPCs.

Public health initiatives focused on maternal, infant, and child health and well-being may have greater potential reach to postpartum mothers. For example, the Maternal, Infant, and Early Childhood Home Visiting Program, which provides in-home support from health care professionals, including nurses, reached over 70,000 pregnant and postpartum women and their families in FY2020.^64^ Home visiting programs, such as this, can provide important extensions of support beyond the traditional postpartum period.

Women also are likely to benefit from counseling by nurse-home visiting by increases in maternal confidence.^65^ In addition, passage of a national comprehensive paid family and medical leave program, such as the proposed American Families Plan,^66^ which would grant up to 12 weeks of paid leave to both parents, could allow for an extension of support for the mother well beyond the first 6 postpartum weeks.

Based on our findings, women who are employed full time, and those with two or more children reported the most frequent experiences of Overload. Extended paid maternity leave policies is a partial solution for employed women. Among countries in the Organisation for Economic Co-operation and Development (OECD),^67^ the United States was the only one without a paid maternity leave policy. The average in OECD countries is 18 weeks of paid leave and in European Union countries is 22 weeks with many countries having additional leave to care for a child at home.^68^

Furthermore, in postpartum education, needs of women having a second or subsequent child are often neglected as they “know the ropes.” For them and other mothers, experiencing high stress, providing community-based parent education, and counseling on stress management may potentially be supported through the community-benefit requirement for nonprofit hospitals.^69^

Fortunately, intervention models for women experiencing postpartum stress have been tested, although all are not successful in achieving stress reduction.^70,71^ Programs classified by Song *et al.* as supportive stress management were found to be especially effective.^70^ For example, a support group intervention focused on maternal transition, stress management, communication, and life planning reduced stress for distressed mothers.^72^

Regarding future research, important questions remain: Are the stressors and coping strategies reported here experienced in more diverse racial and ethnic samples? How are postpartum-specific stressors and coping related to the broader issue to women's health and well-being during the extended postpartum period? Answers to these will be facilitated by continued development of psychometrically strong measures of postpartum-specific research instruments.

### Strengths and limitations

To our knowledge, this is the first study to examine associations between postpartum-specific stressors and coping strategies in the extended postpartum period. Among strengths of this study is the robust sample size for the PCA of categories of postpartum stressors reported by women, which drew on samples from two different regions of the United States. Limitations include low racial/ethnic diversity and the age of the data. The low diversity was a limitation that affected our ability to examine associations between stress and race/ethnicity.

In addition, because our data had limited diversity and were collected before 2000, a limitation is that they may not reflect the postpartum-specific stressors of the more diverse population currently giving birth. For example, from 1990 to (pre-COVID) 2019, the percentage of US births to Hispanic women increased from 14.5% to 23.7%,^73,74^ although employment rates of women at these two time points did remain fairly similar at roughly 57%.^75^ Thus, our findings may not generalize to new mothers today.

To assess if our findings remain relevant to women's lives, we examined recent literature for evidence of their continuance. That evidence supports the view that the stressors we reported continue to be experienced by women. That continuance is likely related to underlying structural factors in the United States affecting women.^1,2,42^

Regarding the SoS Cronbach α values, four of the seven stressor categories met or were near Nunnally's^76^ classic standard of 0.70, but others with less items were likely reduced because scale length is related inversely to reliability. However, we analyzed these as our purposes were exploratory. In addition, the assessment of coping limited women to their top two strategies, and these were subsequently measured dichotomously (used or not used), which limited the sensitivity of measurement. Associations among stressors and coping should be viewed with caution because some may have occurred by chance.

## Conclusions

The 9 to 12 months postpartum time point is a time point when mothers may experience more frequent overload and exhaustion. Overload is most frequent among full time-employed women and those with more than one child. Community resources as well as public health policy and programming to prepare and support women during this challenging interval of the first postpartum year are needed.

## Abbreviations Used

ANOVA: analysis of variance
HPCs: health care providers
KMO: Kaiser–Meyer–Olkin
OECD: Organisation for Economic Co-operation and Development
PCA: Principal components analysis
*SD*s: standard deviations
SoS: Sources of Stress Scale
